# Melanosomal Dynamics Assessed with a Live-Cell Fluorescent Melanosomal Marker

**DOI:** 10.1371/journal.pone.0043465

**Published:** 2012-08-22

**Authors:** Jan M. Bruder, Zachary A. Pfeiffer, Jonathan M. Ciriello, Diana M. Horrigan, Nadine L. Wicks, Benjamin Flaherty, Elena Oancea

**Affiliations:** Department of Molecular Pharmacology, Physiology and Biotechnology, Brown University, Providence, Rhode Island, United States of America; University of Tennessee, United States of America

## Abstract

Melanocytes present in skin and other organs synthesize and store melanin pigment within membrane-delimited organelles called melanosomes. Exposure of human skin to ultraviolet radiation (UV) stimulates melanin production in melanosomes, followed by transfer of melanosomes from melanocytes to neighboring keratinocytes. Melanosomal function is critical for protecting skin against UV radiation, but the mechanisms underlying melanosomal movement and transfer are not well understood. Here we report a novel fluorescent melanosomal marker, which we used to measure real-time melanosomal dynamics in live human epidermal melanocytes (HEMs) and transfer in melanocyte-keratinocyte co-cultures. A fluorescent fusion protein of Ocular Albinism 1 (OA1) localized to melanosomes in both B16-F1 cells and HEMs, and its expression did not significantly alter melanosomal distribution. Live-cell tracking of OA1-GFP-tagged melanosomes revealed a bimodal kinetic profile, with melanosomes exhibiting combinations of slow and fast movement. We also found that exposure to UV radiation increased the fraction of melanosomes exhibiting fast versus slow movement. In addition, using OA1-GFP in live co-cultures, we monitored melanosomal transfer using time-lapse microscopy. These results highlight OA1-GFP as a specific and effective melanosomal marker for live-cell studies, reveal new aspects of melanosomal dynamics and transfer, and are relevant to understanding the skin’s physiological response to UV radiation.

## Introduction

Melanocytes, neural crest-derived cells that produce melanin, reside primarily in the human epidermis and retinal pigmented epithelium [1], [2], but are also found within the cochlea [3], [4], brain [5], [6], and heart [7], [8]. Melanocyte function is critical during embryonic development and throughout life: deficiencies in melanogenesis lead to pigmentation defects and increased susceptibility to melanoma [9], [10], as well as visual and hearing disorders [11], [12].

Cutaneous melanocytes reside on the basal layer of the epidermis and form the epidermal-melanin unit with overlaying keratinocytes [13]. The epidermal unit consists of a single, differentiated, melanocyte that interacts with up to 36 viable keratinocytes. Epidermal melanocytes are responsible for constitutive melanin production, which determines skin color and correlates to melanoma risk [9], [10]. Melanin synthesis relies on the cellular availability of L-tyrosine, which is converted to melanin through a series of reactions catalyzed primarily by the melanosomal enzymes tyrosinase (TYR) and tyrosinase-related proteins (TRP-1 and TRP-2). Melanogenesis is controlled by multiple pathways, including transcriptional regulation and modulation by many signal transduction pathways [reviewed in [14]].

Epidermal melanocytes increase their melanin production in response to solar ultraviolet radiation (facultative pigmentation) [15], [16], a mechanism critical for protecting the skin against UV-induced genotoxic damage [17], [18]. Facultative pigmentation is the result of melanin synthesis in melanocytes, within lysosome-related organelles called melanosomes [[19], reviewed in [20]], followed by melanosomal transfer to surrounding keratinocytes [9], [16], [21]–[27]. In keratinocytes, melanosomes aggreogate in a supranuclear cap that shields the nucleus from UV damage by converting UV photons into heat [28]. Abnormalities in the transfer of melanosomes from melanocytes to keratinocytes result in pigmentation disorders that include Elejalde, Chediak, Higashi, and Griscelli syndromes [29], [30]. While the process of melanin synthesis has been well characterized [20], [31], the mechanism of melanosomal transfer remains largely unknown despite extensive study [24], [32]–[34].

Within melanocytes, melanosomes travel bidirectionally along microtubule tracks, then attach via binding proteins including Rab27a to the distal actin cytoskeleton [[35]–[37], reviewed in [38]], thus accumulating at the cellular periphery at the distal tips. Individual melanocyte processes form multiple cellular contacts with surrounding keratinocytes; melanosomal transfer occurs at these junctions. Based primarily on studies using bright-field time-lapse imaging or electron microscopy and immunolabeling, several models for melanosomal transfer have been proposed: 1) exocytosis/phagocytosis, in which melanosomes are exocytosed by melanocytes and then phagocytosed by keratinocytes [39], [40]; 2) cytophagocytosis, in which keratinocytes internalize a region of melanocytes containing melanosomes [34], [41]; 3) fusion, in which melanocytic filopodia fuse with keratinocytes resulting in transfer [42]; and 4) exosome transfer, in which membrane enclosed exosomes containing one or more melanosomes are released from melanocytes and fuse with keratinocytes [21], [24], [33], [43]. Which of these individual models, or combination thereof, best represents melanosomal transfer in skin has not been conclusively shown, but could be resolved by a suitable fluorescent marker that permits tracking of melanosomes in live cells.

To monitor melanosomal dynamics within melanocytes and their transfer to keratinocytes in real-time, we designed and tested a fluorescent melanosomal marker by fusing green or mCherry fluorescent proteins to the melanosomal proteins Rab27a or Ocular Albinism 1 (OA1). We evaluated the specificity of the fluorescent fusion proteins by colocalization with established immunocytochemical melanosomal markers including TRP-1 and Pmel17. We determined that OA1 fluorescently tagged at the carboxy-terminus is a specific, stable, and effective tool to visualize and quantify melanosomal dynamics in cultured primary human epidermal melanocytes (HEMs). Using OA1-GFP, we show melanosomes are highly dynamic and move non-directionally with variable speeds. Our study reveals that melanosomes can switch between two types of movement, which correspond to restricted diffusion and active transport. We also showed that active transport increases within minutes of exposure to physiological doses of UV.

In addition, we used OA1-GFP-expressing HEMs to image transfer in live co-cultures and found that transfer involves extension and attachment of melanocyte filopodia to keratinocytes, followed by transfer of melanosomes and filopodial retraction. OA1-GFP is a valuable tool for investigating the regulation of melanosomal dynamics by intracellular and extracellular stimuli and for further elucidating the mechanism of melanosomal transfer. Real-time visualization allows for quantitative measurement of melanosomal movement and will provide further insight into the dynamics of organelle trafficking.

## Results

### Design and Generation of Fluorescent Melanosomal Markers

We sought to design a live-cell melanosomal marker by attaching a fluorescent protein tag to a melanosomal-specific protein. We first identified candidate proteins that localize primarily to melanosomes, attach to the melanosomal membrane by either transmembrane domains or a lipid modification, and are oriented such that either one or both termini are cytosolic (to prevent low luminal pH and/or light absorption by melanin from interfering with fluophore efficiency). Based on these criteria, we identified two candidates for a fluorescent melanosomal marker: OA1 [44] and Rab27a [45].

**Figure 1:**
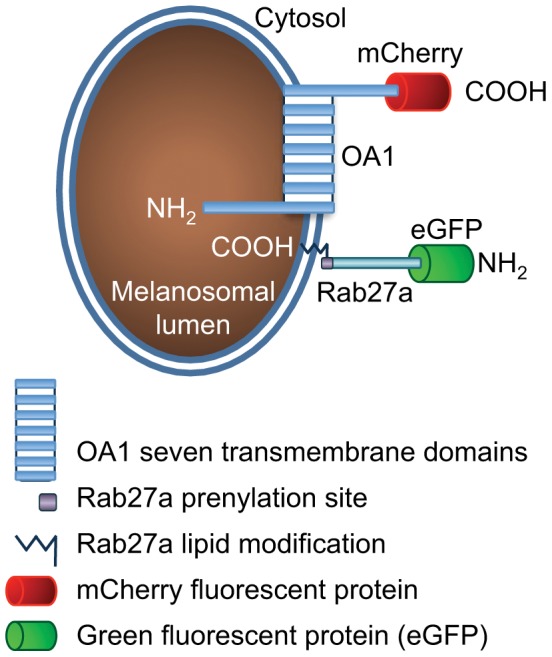
Schematic representation of the fluorescent fusion protein OA1-mCherry and GFP-Rab27.

**Figure 2:**
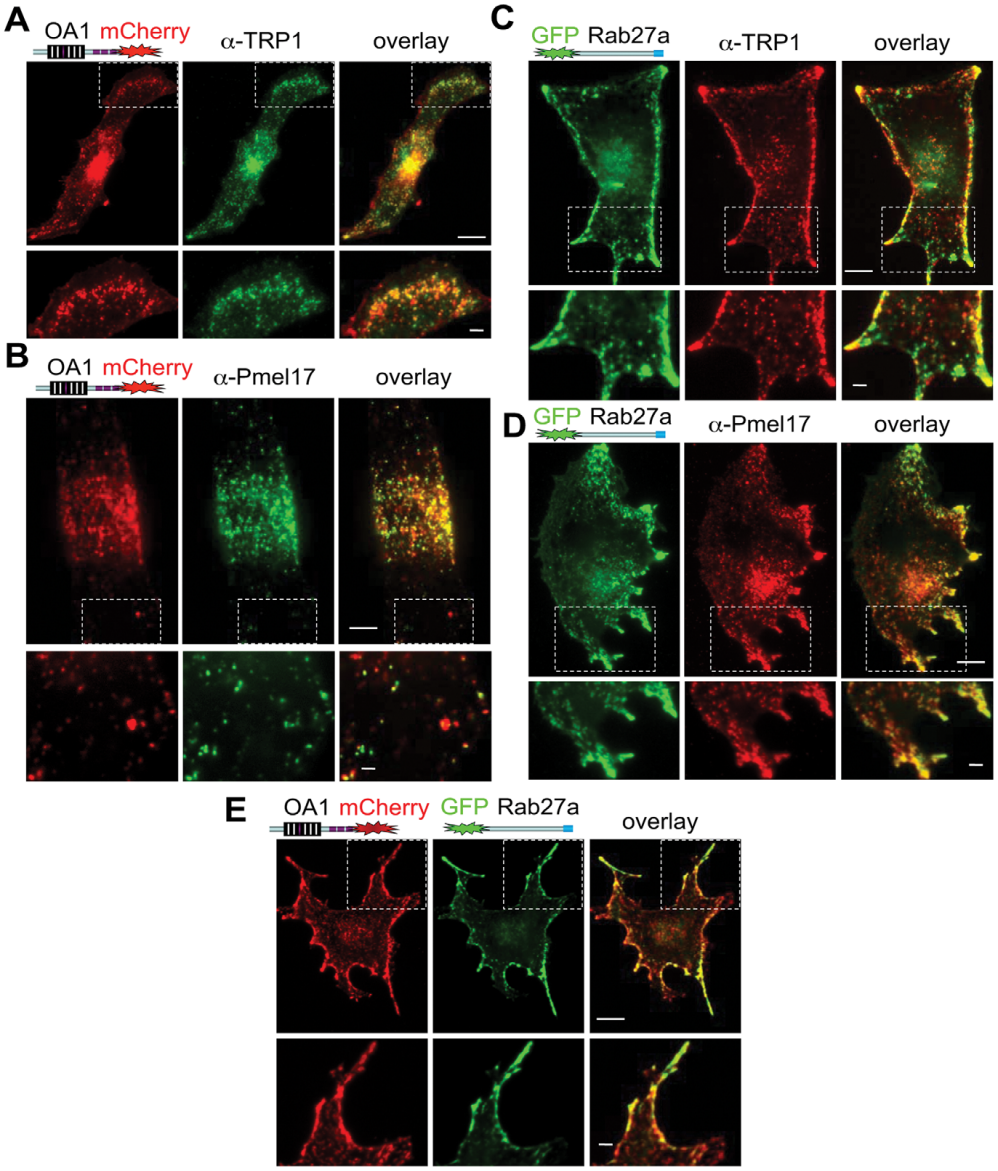
OA1-mCherry and GFP-Rab27a localization in B16-F1 mouse melanoma cells. A. Fluorescent images of B16-F1 cells expressing OA1-mCherry (left panels), immunostained with anti-TRP-1 antibody (middle panels), and shown as overlay (right panels). B. Fluorescent images of B16-F1 cells expressing OA1-mCherry (left), immunostained with anti-Pmel17 antibody (middle), and shown as overlay (right). C. Fluorescent images of B16-F1 cells expressing GFP-Rab27a (left), immunostained with anti-TRP-1 antibody (middle), and shown as overlay (right). D. Fluorescent images of B16-F1 cells expressing GFP-Rab27a (left), immunostained with anti-Pmel17 antibody (middle), and shown as overlay (right). E. B16-F1 cells co-expressing OA1-mCherry (left) and GFP-Rab27a (middle), and shown as overlay (right). Bottom panels show enlarged area marked by white box in the respective top pannel. Calibration bars: 8 µm (top images), 2 µm (bottom images) for all panels.

OA1 is a putative melanosomal G-protein coupled receptor (GPCR) with a luminal amino-terminus and cytosolic carboxy-terminus [44], [46], [47], [48] (**Fig. 1**) that is selectively activated by L-DOPA, a by-product of melanin synthesis [49] and a potential regulator of melanocyte function [reviewed in [50]]. We attached either green (GFP) or red (mCherry) fluorescent protein [51] to the carboxy-terminus of OA1 (OA1-GFP or OA1-mCherry) and tested the cellular localization of these constructs in melanocytes (**Fig. 2**). Rab27a is a small GTPase anchored to the melanosomal membrane by a carboxy-terminal lipid modification. Rab27a functions in melanosome transport by mediating their interaction with distal actin-rich regions of melanocyte processes _ENREF_40 [35], [52], [53], [54]. To test whether Rab27a can function as a melanosomal marker, we fused eGFP to the cytosolic amino terminus of Rab27a (GFP-Rab27a, **Fig. 1**).

### Melanosomal Localization of OA1-mCherry and GFP-Rab27a in Mouse B16-F1 Melanoma Cells

To assess the localization of OA1-mCherry and GFP-Rab27a, we expressed them independently in mouse B16-F1 melanoma cells and used fluorescence microscopy to analyze their colocalization with established antibodies against melanosomal-specific proteins tyrosinase-related protein 1 (TRP-1) and Pmel-17. We quantified the colocalization of the fluorescent fusion proteins and endogenous melanosomal markers using the Mander’s overlap coefficient (R) and the Intensity Correlation Quotient (ICQ) [55]. For R, a value of 1 represents perfect correlation, while the ICQ ranges between −0.5 and 0.5, with the latter reflecting dependent staining.

OA1-mCherry localized to small vesicular structures throughout the cytosol and showed colocalization with anti-TRP-1 and anti-Pmel-17 antibodies (**Fig. 2A, B**). In cells expressing OA1-mCherry, red fluorescence and immunostaining patterns showed increased density of fluorescent vesicular structures within the perinuclear region (**Fig. 2A, B**), as previously noted [56]. Representative R and ICQ values reflect substantial overlap between the localization of OA1-mCherry and TRP-1 or Pmel-17 (**Table 1** **,** for PDM scatter plots see **Fig. S1**).

**Table 1:** Colocalization correlation coefficients for OA1-mCherry, GFP-Rab27a, and TRP-1 or pMel17 in B16-F1 mouse melanoma cells.

	Mander’s Correlation Coefficient - R (max: 1.0)	Intensity Correlation Quotient - ICQ (max: 0.5)
OA1-mCherry vs. TRP-1	0.90	0.46
OA1-mCherry vs. pMel17	0.98	0.45
GFP-Rab27a vs. TRP-1	0.83	0.38
GFP-Rab27a vs. pMel17	0.91	0.45

Mander’s Correlation Coefficient (R) and the Intensity Correlation Quotient (ICQ) for the cells shown in Fig. 2A–D.

Expression of GFP-Rab27a in B16-F1 cells resulted in diffuse cytosolic fluorescence, with visible vesicular structures localized primarily at the cell periphery, likely a result of Rab27a binding to cortical actin [52], [53]. The majority of GFP-Rab27a-containing structures at the cell periphery were also immunolabeled by anti-TRP-1 and anti-Pmel17 antibodies (**Fig. 2C, D**), as illustrated by the R and ICQ values (**Table 1**). The peripheral localization of TRP-1 and Pmel-17 immunolabeled melanosomes (when compared with non-transfected or OA1-mCherry-expressing cells), suggests that exogenous expression of Rab27a alters melanosomal distribution (c.f. **Fig. 2C, D** **;** **Fig. 2A, B**).

To further examine the cellular distribution of OA1-mCherry and GFP-Rab27a, we assessed their colocalization in B16-F1 cells (**Fig. 2E**). In co-transfected cells the majority of fluorescent vesicular structures contained both OA1-mCherry and GFP-Rab27a (**Fig. 2E**). The increased perinuclear fluorescence observed in cells expressing OA1-mCherry alone was attenuated in cells co-expressing GFP-Rab27a, but the peripheral distribution pattern observed in cells with GFP-Rab27a persisted in the presence of OA1-mCherry. The immunolabeling results together with the calculated correlation coefficients for colocalization of OA1-mCherry and GFP-Rab27a suggest both proteins localize primarily to melanosomes in B16-F1 cells. Due to slightly higher correlation coefficients calculated for OA1-mCherry (**Table 1**) and to avoid potential artifacts arising from the anchoring of Rab27a-positive melanosomes to cellular cortical regions, we used fluorescently-tagged OA1 for subsequent studies.

### OA1-GFP Localizes to Melanosomes in Primary Human Melanocytes

**Figure 3:**
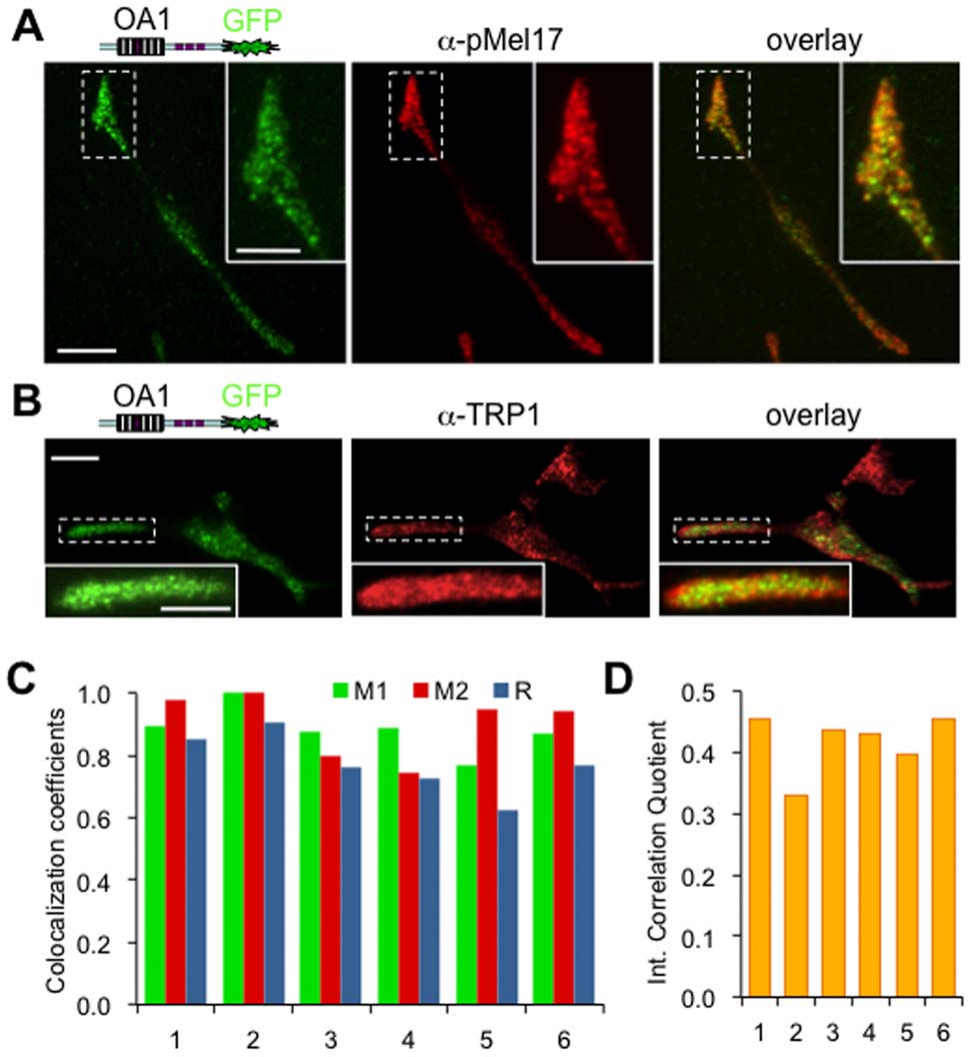
Melanosomal localization of OA1-GFP in primary human melanocytes. A. HEMs expressing OA1-GFP (left), immunostained with anti-Pmel17 antibody (middle), and shown as overlay (right). Calibration bar: 8 µm (image), 2 µm (inset). B. HEMs expressing OA1-GFP (left), immunostained with anti-TRP-1 antibody (middle), and shown as overlay (right). Calibration bar: 8 µm (image), 2 µm (inset). Inset shows the enlarged area marked by the dashed line. C. Colocalization coefficients for representative HEMs expressing OA1-GFP (green) and immunostained with anti-TRP-1 antibody (red). The Mander’s overlap coefficient (M, blue bars) and the split coefficients for the green (M1, green bars) and red (M2, red bars) images have values close to maximal. D. The Intensity Correlation Quotient (ICQ) for six representative HEMs expressing OA1-GFP (green) and immunostained with anti-TRP-1 antibody (red) has values close to maximal value of 0.5.

Towards our goal of imaging melanosomal dynamics and transfer in real-time, we assessed the localization of OA1-GFP in cultured HEMs. Since HEMs are not amenable to conventional DNA transfection methods, we used a lentiviral system to express the fluorescent markers; HEMs expressing OA1-GFP maintained fluorescence over prolonged periods of time (weeks to months). To determine the localization of OA1-GFP in HEMs while minimizing photobleaching during time-lapse acquisition we used objective-based Total Internal Reflection Fluorescent (TIRF) microscopy to selectively illuminate fluorophores within ∼250 nm of the plasma-membrane/glass cover-slip interface. We found that, similarly to B16-F1 cells, OA1-GFP is localized to small vesicular structures in HEMs, the majority of which are immunolabeled by anti-TRP-1 and anti-Pmel17 antibodies (**Fig. 3A, B** **, Fig. S2**).

To quantify the colocalization of OA1-GFP and TRP-1 immunostained with a red fluorescent secondary antibody in HEMs, we calculated Mander’s overlap coefficient (R) and the split colocalization coefficients for the green (M1) and red channels (M2) for six representative cells (**Fig. 3C**). M1 and M2 are normalized against total pixel intensities and avoid issues related to absolute intensities of the green or red signal. Average R was (0.77±0.09), reflecting significant colocalization of OA1-GFP with TRP-1; both M1 and M2 had values close to 1 (**Fig. 3C**), suggesting that most of the OA1-GFP marker is localized to melanosomes. In addition, the calculated ICQ for each cell ranged between 0.33 and 0.46 (**Fig. 3D**, average (0.42±0.04)), indicating that the intensities of the green and red images are dependent. Thus, we conclude that OA1-GFP is an effective live-cell marker to monitor melanosomes in human melanocytes.

### OA1-GFP Labeled Melanosomes are Highly Dynamic

**Figure 4:**
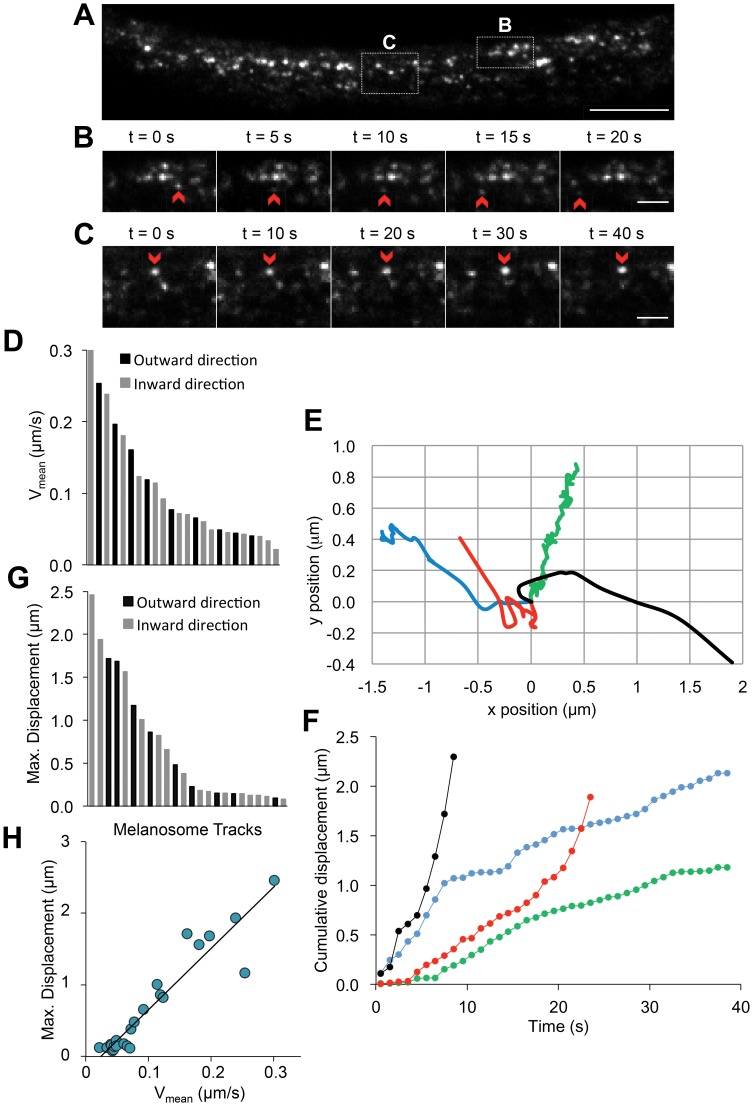
Melanosomal dynamics measured by TIRF imaging of OA1-GFP labeled melanosomes. A. TIRF image of a distal cellular extension of a HEM cell expressing OA1-GFP. Calibration bar: 8 µm. White boxes are expanded and detailed in B and C. A time-lapse movie showing melanosomal movement in this region is available in Movie S1. B. TIRF image series tracking one melanosomes (red arrows) with high average velocity (0.34±0.05 µm/s). (see also Movie S2). Changes in the fluorescence intensity reflect changes in the z-position of the melanosome. Calibration bar: 4 µm. C. TIRF image series tracking one melanosome (red arrows) with low average velocity (0.09±0.01 µm/s). (see also Supplementary Materials, Movie 3). Calibration bar: 4 µm. D. Average velocity of individual melanosomes for particles traveling towards the cell periphery (outward, shown in black) or towards the nuclear region (inward, shown in gray). E. Trajectories of representative melanosomes exhibiting restricted mobility (green trace), fast movement along mostly linear x-y trajectories (black trace) or a combination of the modes of movement. The red trajectory corresponds to a melanosome that initially has restricted movement and then starts moving along a linear trajectory, while the blue trace corresponds to a melanosome that has initial fast and linear movement and then becomes stationary. F. Cumulative displacement of the four representative melanosomes shown in Fig. 4E as a function of time. The slope of the cumulative displacement reflects the speed of the movement. Melanosomes with restricted mobility have a small slope that reflects slow movement (green trace in 4E vs. 4F) and fast melanosomes have a high slope (black trace in 4E vs. 4F). The red trace has an initial small slope (t <20 s) that changes to a high slope due to increased mobility (red trace in 4E vs. 4F), while the blue trace illustrates a melanosome moving fast (t <8 s) and then slowing down (blue trace in 4E vs. 4F). G. The maximal displacement of individual melanosomes for outward- (black) and inward- (gray) moving particles. H. The maximal displacement of individual melanosomes is a linear function of average velocity.

To investigate the dynamics of OA1-GFP-labeled melanosomes with high spatio-temporal resolution, we used TIRF microscopy to image live cells. TIRF microscopy minimizes the amount of light reaching fluorophores, thus minimizing the effects of incident light during quantification of melanosome motility. Series of TIRF images of individual HEMs expressing OA1-GFP acquired every 0.5–1 s over 5–10 min showed that OA1-GFP labeled melanosomes are highly dynamic and travel bidirectionally throughout the cytosol at variable speeds along mostly linear and longitudinal trajectories (**Fig. 4A** **,** **Movie S1**). Two types of melanosomal movement were readily apparent: fast-moving melanosomes which could be followed for short time intervals (<20 s) before moving outside the field of view (**Fig. 4B** **, Movie S2**) and slow-moving melanosomes which could be tracked for longer time intervals (>200 s) (**Fig. 4C** **, Movie S3**).

To quantify melanosomal dynamics using TIRF, we monitored the x-y positions of individual melanosomes as a function of time. Z-directional movement of melanosomes is detected in TIRF as a change in fluorescence intensity; however, since melanosomes become undetectable once they move beyond the TIRF z-range (∼250 nm from the cell-glass interface), we focused on their x-y dynamics. We calculated the average velocity of individual melanosomes (V_avg_) by calculating the mean distance travelled in each 1 s interval over 10 s. Values calculated for 24 melanosomes from >10 cells revealed highly variable average velocities, ranging from 0.02 µm/s to 0.30 µm/s (**Fig. 4D** **)**.

To determine what types of movement might account for such highly variable velocities, we analyzed the two-dimensional trajectories of individual melanosomes as a function of time **(** **Fig. 4E, F** **).** We found that slow-moving melanosomes exhibit restricted mobility and tend to oscillate around a central position, consistent with them being docked to cytoskeletal structures or the plasma membrane (**Fig. 4E**, green trace, V_avg_ = 0.009 µm/s). In contrast, fast-moving melanosomes travel along mostly linear x-y trajectories (**Fig. 4E**, black trace, V_avg_ = 0.051 µm/s), consistent with active transport along microtubule tracks. Melanosomes with intermediate V_avg_ values (**Fig. 4D**) typically exhibit mixed trajectories: some display initially fast and linear movement and then become stationary (**Fig. 4E**, blue trace, V_avg_ = 0.041 µm/s), while others begin in a stationary position and then move more quickly along a linear trajectory (**Fig. 4E**, red trace, V_avg_ = 0.028 µm/s).

To further analyze the types of melanosomal movement, we represented the cumulative displacement of the melanosomes shown in **Fig. 4E** as a function of time (**Fig. 4F**). The slope of the graphs reflects melanosomal speed, which is consistently slow for the green trace and consistently fast for the black trace, in agreement with the V_avg_ values for these melanosomes. In contrast, the red trace corresponds to a melanosome that switches from slow to fast movement, as illustrated by the increased slope at t >20 s, and the blue trace corresponds to a melanosome that switches from fast to slow movement, as illustrated by the smaller slope at t >8 s. We conclude that oscillatory (stationary) and linear trajectories account for slow and fast V_avg_ values, respectively, while intermediate V_avg_ values result from transitions between different trajectories and depend on the relative duration of the types of movement involved (**Fig. 4F**).

We also quantified the maximal displacement of individual melanosomes as the furthest distance from starting point reached within 10 seconds. Displacements ranged from 0.08–2.46 µm (n = 24) (**Fig. 4G**). A significant number of melanosomes do not travel far from their initial position (50% of melanosomes exhibit displacements within the ((0.08–0.22) µm range), consistent with them being docked to cytoskeletal elements. Melanosomes exhibiting larger maximal displacements are less restricted and travel variable distances.

Our observation that melanosomes exhibit three types of movement − fast and linear, slow and oscillatory, or a combination of the two − predicts that each type of movement will generate a certain range of displacements. For each melanosome, the magnitude of displacement will depend on the length of time it spends displaying each type of movement. To test this prediction, we calculated maximal displacement as a function of average velocity for individual melanosomes (**Fig. 4H**). The linear relationship is consistent with slow-moving melanosomes achieving small displacements, fast-moving melanosomes traveling longer distances, and melanosomes displaying both fast and slow movement achieving displacements proportional to their velocity.

We then examined the directionality of melanosomal movement and found that most individual melanosomes move unidirectionally on longitudinal tracks extending between the perinuclear region and the distal tips. Occasionally melanosomes displayed short, lateral jumps, after which they continued the longitudinal movement along a parallel trajectory (observed for 2 out of 41 melanosomes). We classified directionality as either “outward” (towards the cell periphery) or “inward” (towards the perinuclear region). To determine if directionality is correlated with either velocity or displacement, we graphed V_avg_ or maximal displacement of individual melanosomes classified as either outwardly-moving or inwardly-moving (**Fig. 4D, G**). Since we observed similar distributions of V_avg_ and maximal displacements for both outwardly- and inwardly-moving melanosomes, we conclude that similar dynamics exist in both directions under steady state conditions.

### Ultraviolet Light Induces Changes in Melanosomal Movement

To investigate whether melanosomal dynamics in HEMs is modulated by physiological doses of UV light [15], we used mean square displacement (MSD) to characterize melanosomal motion in living cells. The time dependence of MSD can be used to indicate type of movement: linear trajectories correspond, depending on their slope, to simple (slope = 1) or restricted (slope <1) diffusion; nonlinear trajectories with positive curvatures correspond to directed movement, while nonlinear trajectories with negative curvature correspond to confined movement [57], [58]. We used series of TIRF images to track individual melanosomes before or after UV exposure and calculated the corresponding MSD as a function of time.

**Figure 5:**
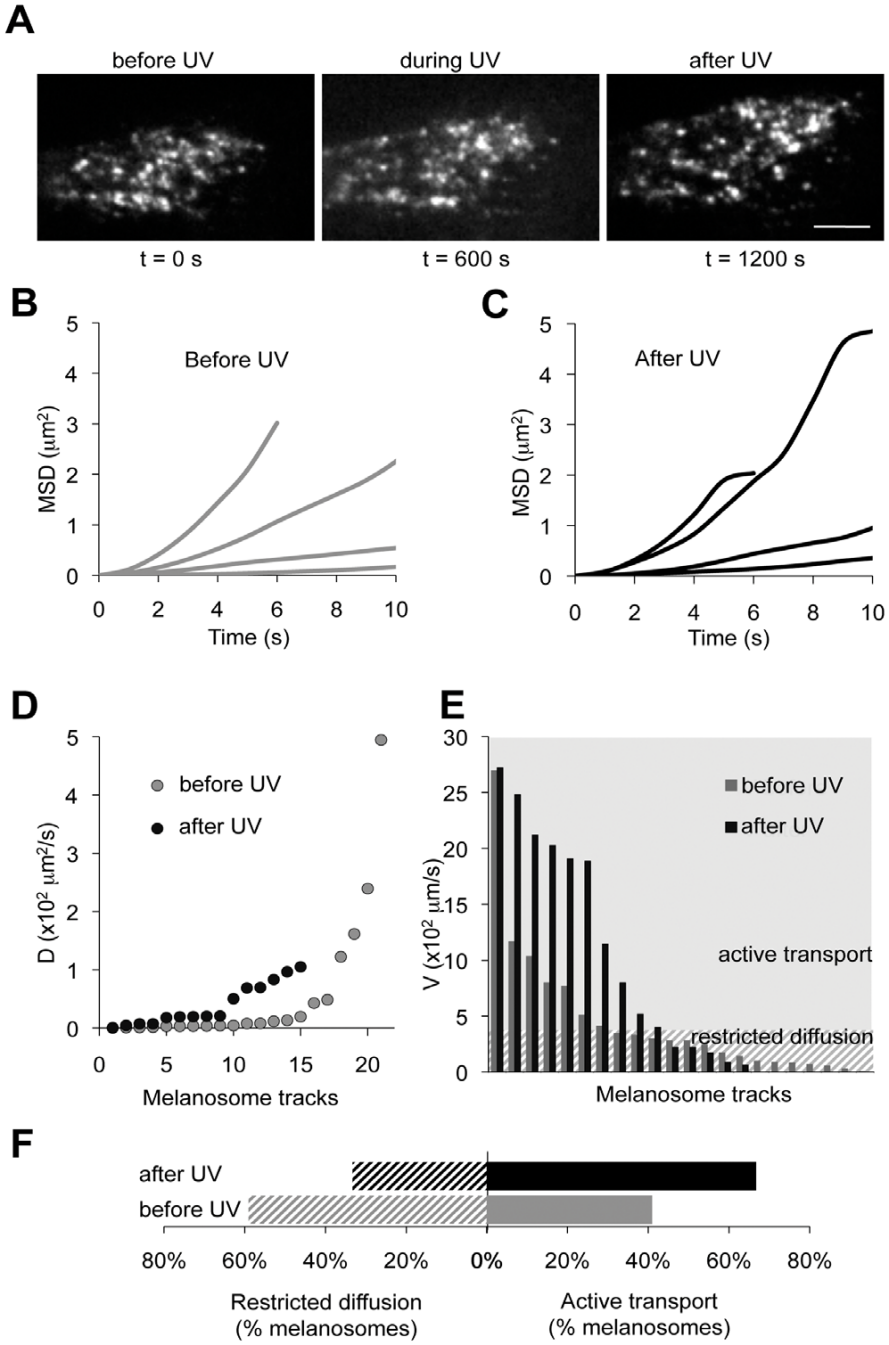
Changes in melanosomal dynamics in response to UV. A. TIRF images of the distal end of a HEM expressing OA1-GFP before (t = 0 s), during (t = 600 s) and after (t = 1200 s) stimulation with UV (200 mJ/cm^2^). Calibration bar: 4 µm. B. Representative Mean Square Displacement (MSD) calculated by tracking individual melanosomes before UV stimulation and represented as a function of time. C. Time dependence of MSD for representative melanosomes imaged after exposure to UV. D. Distribution of diffusion coefficients (D) of individual melanosomes tracked before (gray; n = 22) or after (black; n = 15) UV exposure. E. Mean velocities of melanosomes before (gray) and after (black) UV exposure. Velocities <0.03 µm/s are considered negligible and the corresponding melanosomes move primarily by restricted diffusion (hatched area). Velocities >0.03 µm/s correspond to melanosomes moving by both diffusion and directed movement (gray area). F. In response to UV exposure the fraction of melanosomes moving by active transport increases (black vs. gray bars with V >0.03 µm/s in Fig. 5E), while the fraction of melanosomes moving by restricted diffusion decreases (black vs. gray bars with V <0.03 µm/s in Fig. 5E). 67% of the melanosomes in Fig. 5E move by active transport after UV exposure compared to 41% in unstimulated HEMs (solid black bar vs. solid gray bar) and 33% of the melanosomes in Fig. 5E have restricted diffusion after UV exposure, compared to 59% of melanosomes before UV exposure (hashed black bar vs. hashed gray bar).

We exposed HEMs to physiological UV doses using a setup that permits simultaneous live TIRF imaging of cultured cells and stimulation with irradiances comparable to solar UV [59]. Each experiment consisted of a glass coverslip with HEMs expressing OA1-GFP; all cells were exposed to UV (20 s at 20 mW/cm^2^) corresponding to 400 s full sun exposure on a day with a UV Index of 10, and TIRF images acquired at 1 s intervals before and up to 600 s after UV irradiation. Cell morphology and overall distribution of melanosomes did not significantly change during or <10 min after UV exposure (**Fig. 5A**).

We analyzed the MSD time-dependence (n = 37 melanosomes from 4 independent experiments) before (n = 22) and after (n = 15) UV irradiation. MSD had a linear dependence with slope <1, indicating restricted diffusion, as well as a nonlinear dependence corresponding to directed movement, likely due to active transport of melanosomes (**Fig. 5B, C**). In addition, melanosomes monitored after UV exposure exhibited a significant number of biphasic trajectories with an nonlinear phase followed by a linear phase of smaller slope, corresponding to restricted movement (**Fig. 5C**) [**57**]. This biphasic time dependence is consistent with initial directed movement followed by restricted mobility, and suggests that UV exposure promotes docking of melanosomes to immobile cellular structures, possibly in preparation for transfer to keratinocytes.

To further analyze UV-dependent changes in melanosomal dynamics, we assumed melanosomes undergo diffusion (with a diffusion coefficient D) as well as directed movement (with velocity V). We fit each MSD curve using the formula: MSD(t) = 4Dt + (Vt)^2^, where D is the diffusion coefficient and V the melanosomal velocity [60]. Diffusion coefficients calculated for individual melanosomes were not significantly different before and after UV exposure (**Fig. 5D** **,** before UV: D_avg_ = (0.93±0.44)×10^−2^ µm/s, after UV: D_avg_ = (0.39±0.09)×10^−2^ µm/s; p = 0.9); however, velocity was significantly increased after UV exposure (**Fig. 5E**). Trajectories with small V values can be considered linear (since at small V values (Vt)^2^ in equation (1) becomes negligible) and thus reflect restricted diffusion. We assumed that melanosomes with trajectories fit by V <3×10^−2^ µm/s were linear and move by restricted diffusion. Conversely, melanosomes with V >3×10^−2^ µm/s move primarily by directed movement, likely due to active transport (**Fig. 5E**). Using the graph shown in Fig. 5E, we calculated the fraction of melanosomes displaying each type of movement before and after UV exposure. We found that under steady state conditions, the majority of melanosomes moved by restricted diffusion (59% by restricted diffusion and 41% by directed movement), while after UV exposure an increased fraction began moving by directed movement (66% by directed movement, 34% by restricted diffusion) (**Fig. 5F**).

### Live Melanosomal Transfer in Melanocytes and Keratinocyte Co-culture

**Figure 6:**
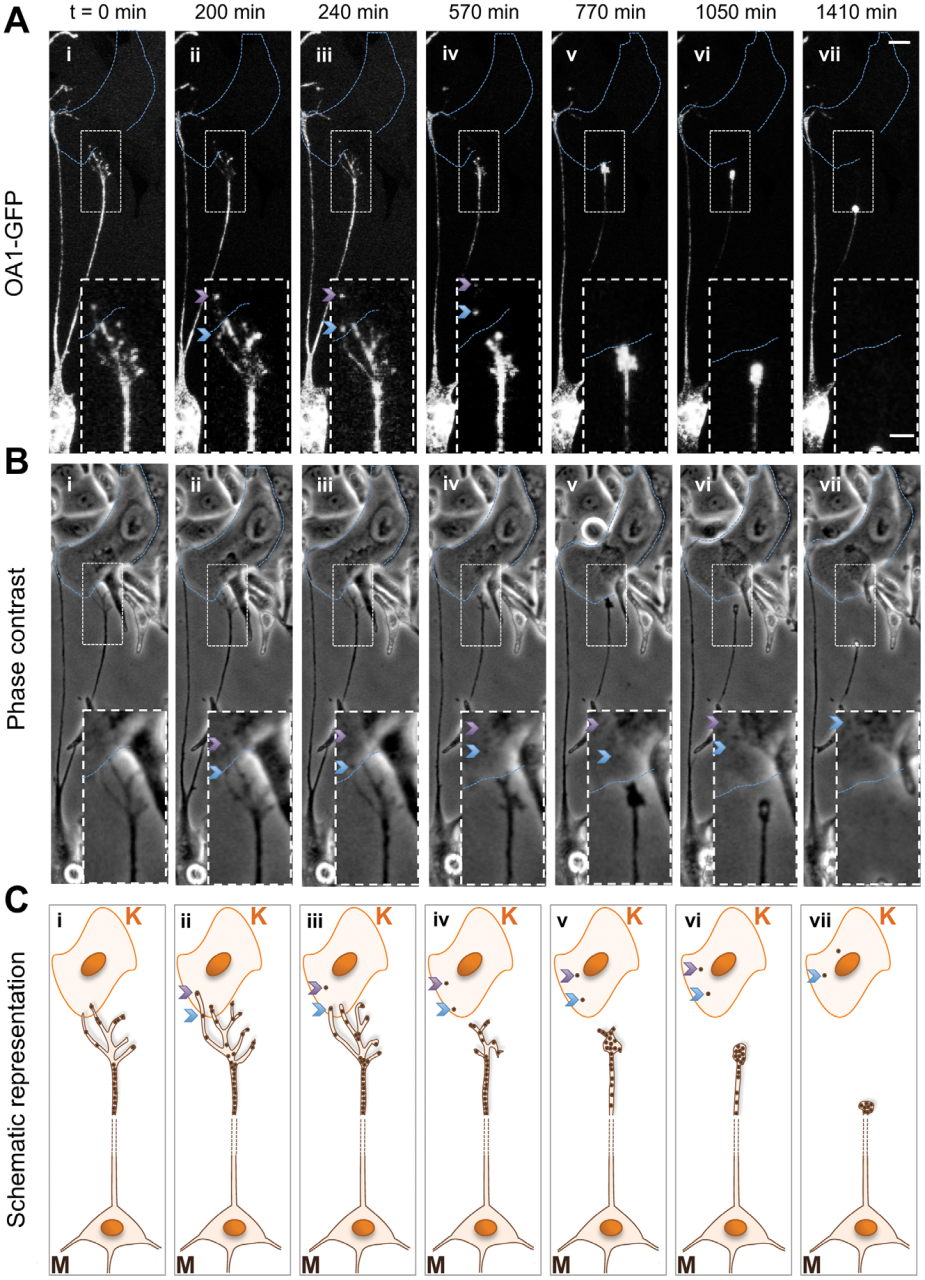
Melanosomal transfer imaged with OA1-GFP in live melanocyte/keratinocyte co-cultures. 1. Fluorescent images of melanosomal transfer in co-cultured OA1-GFP-expressing HEMs and HEKERs showing: (i) contact between a HEM cellular process and an HEKER cell body; (ii, iii) sequential detachment of two melanosomes from the HEM followed by transfer to the HEKER (ii, iii), and retraction of the cellular process (iv, v, vi). Insets show enlarged area depicted by the white dashed square. The arrows indicate positions of transferred melanosomes, Calibration bar: 20 µm (image), 8 µm (inset). 2. Corresponding phase contrast images of the co-cultured cells shown in (A). Insets show enlarged area depicted by the white dashed square. Arrows indicate positions of transferred melanosomes. 3. Schematic representation of the melanosomal transfer process shown in (A) and (B). Cartoon images show: (i) at t = 0 min, a HEM (M) cellular process containing melanosomes extends filopodia towards an HEKER (K); over the following 200 min, a filopodium extends over the HEKER plasma membrane, as more melanosomes are transported along all filopodia; (iii) the tip of the extended filopodium containing melanosomes (purple arrow) becomes detached from the HEM and continues to move within the HEKER; (iv) a second melanosome within the same filopodium (blue arrow) is then transferred to the HEKER as all filopodia begin to retract; (v, vi) filopodia retract over the next ∼500 min; (vii) finally, the cellular process retracts towards the HEM cell body. The two melanosomes remain associated with and continue to move within the HEKER. Time-lapse movies of phase and fluorescent images are available as Movie S4.

We next imaged real-time transfer of fluorescently labeled melanosomes using co-cultures of OA1-GFP-expressing HEMs and primary human epidermal keratinocytes (HEKERs). HEKERS were pre-seeded 24 h before addition of HEMs to prevent HEM-HEKER aggregation during seeding. Co-cultures were imaged 3 h after addition of HEMs. We collected fluorescence and phase contrast images at 10 min intervals for 5 days, while incubating co-cultures at 37°C and 5% CO_2_. Under these conditions, melanosomal transfer is a rare but detectable event (**Fig. 6** **, Movies S4 and S5**).

In a typical recording of melanosomal transfer, we first identified melanosomes based on OA1-GFP expression in a cellular process morphologically resembling a growth cone, which extended from an OA1-GFP expressing HEM towards an HEKER (**Fig. 6i**). As this extension approached the HEKER, thin filopodia containing melanosomes were extended from its tips (**Fig. 6ii**). Upon filopodium-HEKER contact, we observed transfer of a melanosome from the filopodium to the HEKER (purple arrow in **Fig. 6ii** **, iii**). After this initial transfer event, all filopodia began retracting, and remaining melanosomes appeared to be transported in a retrograde manner towards the HEM cell body (c.f. filopodial fluorescence in **Fig. 6A** **iii;** **6Aii**). After partial retraction, the same filopodium established a secondary HEKER connection, transferred a second melanosome, and continued retracting (blue arrow in **Fig. 6iii** **, iv**). Following transfer, the HEM cellular process and filopodia retracted towards the cell body (**Fig. 6iv–vii**), and the transferred melanosomes moved slowly away from the transfer site, towards the interior of the HEKER (**Fig. 6v–vii**). These results suggest that transfer of melanosomes from melanocytes to keratinocytes involves melanocyte filopodia extension, filopodia contact with a keratinocyte, followed by melanosomal transfer and filopodia retraction.

## Discussion

Our results establish fluorescently-tagged OA1 as an effective melanosomal marker for live imaging in melanocytes and enabled us to measure melanosomal dynamics within melanocytes. Using OA1-GFP in melanocyte-keratinocyte co-cultures allowed us to monitor melanosomal transfer in real-time and provided further evidence for a role of melanocyte filopodia in this process [42], [61], [62]. Previous studies reporting detection of melanosomal transfer have relied on bright field microscopy, which does not permit differentiation between melanosomes and other dense organelles, or relied on immunostaining of fixed cells, which does not allow for dynamic analysis or detection of transfer in real-time [34], [42], [63]. Fluorescently tagged OA1 could be instrumental for further elucidating the mechanism of melanosomal transfer and investigating the regulation of melanosomal dynamics by various signaling pathways.

### Developing a Fluorescent Melanosomal Marker for Live-cell Imaging

The development of an effective tool for the study of melanosomes has been hindered by several technical challenges. Melanin is an electron-dense, strongly light-absorbing, and chemically robust substance that readily converts incident light from a large spectrum of wavelengths into heat [64]. While this property makes melanin a very effective UV filter for the skin, it poses a challenge to the development of useful fluorescent melanosomal markers, as fluorophores in the luminal compartment are effectively shielded from receiving or emitting light. Earlier attempts to fluorescently label melanin or melanosomes have thus struggled with obtaining favorable signal to noise ratios [32]. Alternate labeling methods, including radioactive labeling, lacked the temporal and spatial specificity necessary to distinguish melanosomes from other dense cellular vesicles [24], [32], [42]. In this study, we set out to develop, test, and characterize a specific, stable, and efficient fluorescent melanosomal marker for use in studies investigating melanosome dynamics and transfer.

### Localization of Fluorescently Labeled Melanosomes in Melanocytes

In developing a melanosomal marker for real-time imaging, we sought to preserve the cellular distribution and mobility of melanosomes. The high degree of colocalization between OA1-mCherry and GFP-Rab27a with anti-TRP-1 and anti-Pmel17 antibodies confirmed that both fluorescent markers localize to melanosomes. However, Rab27a-GFP expression caused clustering of melanosomes at the cell periphery (**Fig. 2C, D**), an effect likely resulting from melanophilin-mediated binding of Rab27a to cortical actin [**35**], [36], [**65**]. Our ability to monitor and quantify melanosomal dynamics and transfer using OA1-GFP-expressing melanocytes suggests that OA1 overexpression does not significantly disrupt melanosomal distribution, motility, or function, as indicated by transfer of OA1-GFP-tagged melanosomes from HEMs to HEKERs in primary human cell culture.

### Tracking Fluorescently Labeled Melanosomes in Melanocytes and Co-cultures

Melanosomes move along microtubules using motor proteins and become bound to cortical actin structures at the distal tips of melanocyte processes (reviewed in [66]). However, key aspects of their movement, like velocity, trajectory, and diffusion coefficient, have not been determined, primarily due to lack of technical tools. Our OA1-GFP assay allowed us to observe and quantify the dynamics of individual melanosomes with high temporal resolution (<1 s) using TIRF microscopy. We quantified melanosomal movement along the x- and y-axis by single particle tracking using TIRF image series; our study reveals that melanosomes are highly dynamic (**Fig. 4**), move with a wide range of average velocities (**Fig. 4D**) and have different types of trajectories (**Fig. 4E**).

The wide range of average velocities is consistent with the notion that melanosomes move with a combination of fast and slow kinetics. We propose that linear trajectories associated with faster movement correspond to motor protein-mediated movement of melanosomes along microtubules, whereas slow or corralled movement corresponds to melanosomes anchored to either cytosolic or cortical actin structures. We also found that melanosomes can transition between fast and slow kinetics reversibly; trajectory analysis and the time-dependencies of MSDs (**Fig. 5B, C**) showed that melanosomes with fast, linear movement can become slow-moving, while melanosomes with restricted movement could “escape” and begin to move more quickly. Under our experimental conditions such transitions were infrequent, but the molecular mechanisms they rely on is likely to be important for melanosomal function.

OA1-GFP also permitted real-time monitoring of melanosomal transfer. Our observations, which support previous results [34], [42], [63], showed that contact between melanocytes and keratinocytes is dynamic and mediated by melanosome-containing filopodia that form cellular contacts with keratinocytes prior to transfer. Time-lapse recordings show that melanocytes actively explore their environment and make numerous transient contacts with surrounding cells up to a few hundred microns away. In contrast, keratinocytes are far less dynamic and do not initiate cell-cell contacts.

In conclusion, OA1-based fluorescent markers are valuable tools for the study of intracellular and intercellular melanosomal movement and transfer and will lead to a better understanding of organelle dynamics. Such markers will prove helpful for characterizing the molecular mechanisms mediating melanosomal dynamics, the understanding of which could reveal new regulatory pathways critical to skin physiology.

## Methods

### Cell Culture and Transfection

All cells were maintained in a humidified incubator at 37°C and 5% CO2 and received fresh media every 3 days. Primary human neonatal epidermal melanocytes (HEMs; Invitrogen, Carlsbad, CA) were maintained in Medium 254 supplemented with PMA-free Human Melanocyte Growth Supplement (HMGS-2, Invitrogen) and penicillin/streptomycin (100 U/ml, Invitrogen). Cells were maintained in culture for <20 passages. Primary human keratinocytes (HEKERs; Invitrogen) were maintained in EpiLife Medium supplemented with Human Keratinocyte Growth Supplement (HKGS, Invitrogen) and penicillin/streptomycin (100 U/ml). For HEM/HEKER co-cultures, ∼3×10^5^ HEKERs were seeded into each well of 6-well plates, 16–24 h before adding to each well 6×10^4^ HEMs expressing OA1-GFP. HEMs were first trypsinized (0.25%, Invitrogen), treated with trypsin inhibitor (0.05%, Invitrogen), resuspended in HEKER media, counted, and then added to each well.

Mouse B16-F1 melanocytes were cultured in medium containing DMEM, 10% FBS (Gibco/Invitrogen), and penicillin/streptomycin (100 U/ml). Human Embryonic Kidney 239T cells (HEK 293T; ATCC, Manassas, VA) used for lentiviral production were cultured in Dulbecco’s Modified Eagle Medium (DMEM, Invitrogen) supplemented with 10% fetal bovine serum (FBS, Atlanta Biologicals, Lawrenceville, GA) and penicillin/streptomycin (100 U/ml). For viral production HEK 293T cells were maintained in antibiotic-free medium, or in medium supplemented with 20% bovine serum albumin (BSA, Sigma, St. Louis, MO). B16-F1 and HEK 293T cells were transfected using Lipofectamine 2000 (Invitrogen), according to manufacturer’s protocol.

### Subcloning of Fluorescently Tagged Proteins

Human OA1 cDNA (GPR143, NM_000273.2) kindly provided by Dr. S. Orlow (New York University, New York) was amplified and subcloned into EcoR1/Xho1 restriction sites of pcDNA4/TO vector (Invitrogen) containing inframe GFP or mCherry coding sequence 3′ of the target restriction sites. Human Rab27a cDNA (NM_004580.3) was amplified from HEMs by RT-PCR using the following forward and reverse primer pair: 5′CATCATAAGCTTGGAGCTGGTGCAATGTCTGATGGAGATTATGATTACC and 5′CATCATGGATTCTCAACAGCCACATGCCCC3’. The resulting PCR product was cloned into HindIII/BamHI sites of pcDNA4T/O containing GFP sequence inframe and 5′ of the cloning sites. Lentiviral constructs were generated by recombination of GFP-OA1 or mCherry-OA1 coding sequence into pDONR and then pLenti4/TO-V5-DEST vectors using Gateway BP/LR Clonase (Invitrogen) according to the manufacturer’s protocol. Insertion of the correct sequences of interest into the respective plasmids was confirmed by DNA sequencing (Macrogen Inc., Rockville, MD).

### Lentiviral Production and Transduction

Viral particles were produced as described elsewhere [67]. Briefly, 6 cm culture dishes of 80% confluent HEK 293T cells were switched to antibiotic-free media 24 h before transfection. HEK 293T cells were transfected using TransIT-2020 transfection reagent (Mirus Bio LLC, Madison, WI) with pcDNA-OA1-GFP, VSV-G, and pCMVdR8.91 plasmids [68] at a 3∶2:1 ratio. 3 h after addition of transfection mix, cells were switched to high BSA media. Supernatant containing viral particles was harvested at 24 h and 48 h post-transfection, pooled and cellular debris was removed by centrifugation (500 rpm, 5 min). Viral particles were concentrated from decanted supernatant using a Lenti-X Concentrator kit (Clontech, Mountain View, CA) according to manufacturer instructions. The resulting pellet was re-suspended in 1 ml of sterile PBS and used for transduction; remaining stock was frozen and maintained at −80°C. Low passage (<3) HEMs at 60% confluence in 35 mm culture dishes were transduced with 200 µl viral stock. Transduction efficiency was assessed via fluorescence microscopy after 7 days. Expression of OA1-GFP was stable over >4 weeks and several passages.

### Immunostaining

Cells plated on glass coverslips were rinsed with phosphate buffered saline (PBS) and fixed with 4% paraformaldehyde (PFA) for 10 min, treated with 10 mM ammonium-chloride solution (Sigma-Aldrich) for 10 min to reduce autofluorescence, followed by permeabilization with 0.1% triton X-100 (Sigma-Aldrich) for 5 min, and incubation with blocking solution (10% normal goat serum, 1% BSA in PBS) for 1 h at RT. Primary antibodies in blocking solution [mouse-anti-TRP-1 (clone TA-99, Covance Inc., Princeton, NJ), 1∶50 dilution and mouse anti-Pmel17 (clone HMB-45, Dako, Carpinteria, CA), 1∶30 dilution were applied to the samples for 1 h at RT, followed by secondary antibodies (Alexa Fluor, Molecular Probes/Invitrogen) for 1 h at RT.

### Image Acquisition, Processing, and Colocalization Analysis

Fluorescence and TIRF microscopy were carried out on an inverted fluorescent Olympus IX71 microscope (Olympus America Inc., Center Valley, PA) with two-color TIRF assembly and filter sets for GFP or mCherry emission. TIRF illumination was achieved with 473 nm and 561 nm diode-pumped lasers (CrystaLaser, Reno, NV). Images were captured using MetaMorph (Molecular Devices, Downington, PA, USA). Pseudo-colored overlays were generated using solid color layer overlays and linear burn functions in Adobe Photoshop CS4 (Adobe, San Jose, CA); layers were stacked using the difference layer blend mode.

Colocalization analysis was performed using the ImageJ Intensity Correlation Analysis plugin: http://www.uhnresearch.ca/facilities/wcif/imagej/colour_analysis.htm.

We used Mander’s Overlap coefficient (M), Mander’s split coefficients for the green (M1) and red (M2) channels, and the Intensity Correlation Quotient (ICQ). M analysis requires that the number of objects in both channels to be approximately equal; it ranges between 0 and 1, with 1 being high colocalization. M1 and M2 are Mander’s coefficients calculated for each channel and reflect how well each channel overlaps the other, while avoiding issues relating to absolute intensities of the signal, since they are normalized against average pixel intensity. ICQ ranges between −0.5 and 0.5 and indicates whether the intensity of the green and red pixels varies synchronously for corresponding x,y locations in two images.

Colocalization scatter plots were obtained by representing pixel intensity (y-axis) vs. the Product of the Differences from the Mean (PDM) on the x-axis, where:
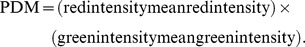



Plots were created using the imageJ Intensity Correlation Analysis plugin [55] with thresholds set to zero. The PDM method analyzes the variance of pixel intensity compared to the mean intensity of each channel as a whole and is thus normalized to the average brightness of each channel. Positive PDM values correspond with colocalized pixels in both channels. Negative values indicate pixels that do not co-vary in their intensity in both channels and are not colocalized.

HEM-HEKER co-cultures were imaged over 5 days using a Nikon TE 2000 inverted fluorescence microscope with a custom stage incubator, humidification, and CO_2_ assembly (Solent Scientific, Segensworth, UK). Using a motorized stage and focus drive, phase contrast and GFP images of up to 10 independent x-y positions were captured at 10 min intervals in 4 z-planes spanning 20 µm. An optimal focal plane was visually selected and image series were converted to 8-bit tiffs using Volocity LE (x64) v5.3.1 (Improvision/PerkinElmer, Waltham, MA) and stack registered using the Image Stabilizer plugin in ImageJ (http://www.cs.cmu.edu/~kangli/code/Image_Stabilizer.html).

### Melanosomal Dynamics and Mean Square Displacement Analysis

OA1-GFP-positive melanosomes were tracked using algorithmic Track Object feature of MetaMorph. The x-y coordinates at each time point were used to calculate the average velocity and maximal displacement for each melanosome. Cumulative displacement was calculated using the formula:
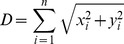



The mean square displacement (MSD) was calculated for individual melanosomes at each time point (n) in Matlab (MathWorks, Natick, MA) using the following formula [69]:
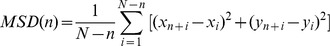
where N is the total number of time points collected, (x_n_, y_n_) is melanosome position at time point (n). Particle mobility was quantified by determining the diffusion coefficient (D) and velocity (V) of each particle, calculated by fitting the first 10 points of the MSD vs. time data with the equation:



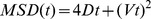



#### Statistical analysis

Numerical data are presented as mean ± s.e.m. P-values were calculated by Student’s *t*-test and considered significant when p<0.05.

## Supporting Information

Figure S1 **Scatter plots showing colocalization of fluorescent pixels from green (channel 1) and red (channel 2) channels in B16-F1 cells from the corresponding panels in** **Fig. 2** **, quantified in** **table 1** **.** Left and middle columns contain graphs that plot pixel intensity (y-axis) versus the Product of the Differences from the Mean (PDM) value (x-axis) (see Methods). Positive PDM values correspond with colocalized pixels in both channels. Negative values indicate pixels that do not co-vary in their intensity in both channels and are not colocalized. The graphs in the right column plot pixel intensity of channel 1 versus pixel intensity of channel 2 as an additional means of relating the brightness data in both channels. The data in these graphs are not normalized.
(TIF)

Figure S2 **Scatter plots showing colocalization of fluorescent pixels from red and green channels in HEMs from corresponding sub-figures in** **Fig. 3** **, quantified in the text.** Same setup, procedure, and layout were used as described in S1.
(TIF)

Movie S1 **TIRF imaging of OA1-GFP labeled melanosomes.** TIRF time-lapse imaging of melanosomal movement within a distal cellular extension of a HEM expressing OA1-GFP. Images were acquired at 1 s intervals and shown at 10 frames per second. Calibration bar: 8 µm. White boxes are expanded and detailed in Movies S2 and S3.
(MOV)

Movie S2 **TIRF imaging of fast-moving OA1-GFP labeled melanosomes.** TIRF time-lapse tracking of one melanosome (arrow) with high average velocity (0.34±0.05 µm/s). Changes in the fluorescence intensity reflect changes in the z-position of the melanosome. Images were acquired at 1 s intervals and shown at 10 frames per second. Calibration bar: 4 µm.
(MOV)

Movie S3 **TIRF imaging of slow-moving OA1-GFP labeled melanosomes.** TIRF time-lapse tracking of one melanosome (arrow) with low average velocity (0.09±0.01 µm/s). Images were acquired at 1 s intervals and shown at 10 frames per second. Calibration bar: 4 µm.
(MOV)

Movie S4 **Melanosomal transfer imaged with OA1-GFP in live melanocyte/keratinocyte co-cultures.** HEMs expressing OA1-GFP are shown as fluorescence time-lapse series in panels C and D and as synchronized phase fluorescent time-lapse series are shown in panels A and B. Panels B and D are 200% enlargements of the regions marked with a dashed box in the first two seconds of panels A and C, respectively. Melansomes are visible as dark particles in phase contrast and as fluorescent particles in fluorescence imaging. Two melanosomal transfer events are shown between the melanocyte marked M and the keratinocyte marked K. Images were acquired at 10 min intervals and shown at 10 frames per second. Scale bar = 20 µm.
(MOV)

Movie S5 **Phase contrast (left) and fluorescence time-lapse (right) movie of HEMs expressing OA1-GFP and HEKERs in co-culture.** Zoomed-out view of HEM/HEKER co-culture shows that HEMs are highly dynamic cells that actively explore their environment. While HEMs make contact with several HEKERs over the duration of the movie, HEKERs appear to be mostly stationary. Images were acquired at 10 min intervals and played at 30 frames per second. Scale bar = 20 µm.
(MOV)
