# Supplementary figures and images for: Melanosomal Dynamics Assessed with a Live-Cell Fluorescent Melanosomal Marker
Source: PLoS One. 2012 Aug 22;7(8):e43465. doi: 10.1371/journal.pone.0043465 (PMC3425493; doi:10.1371/journal.pone.0043465)

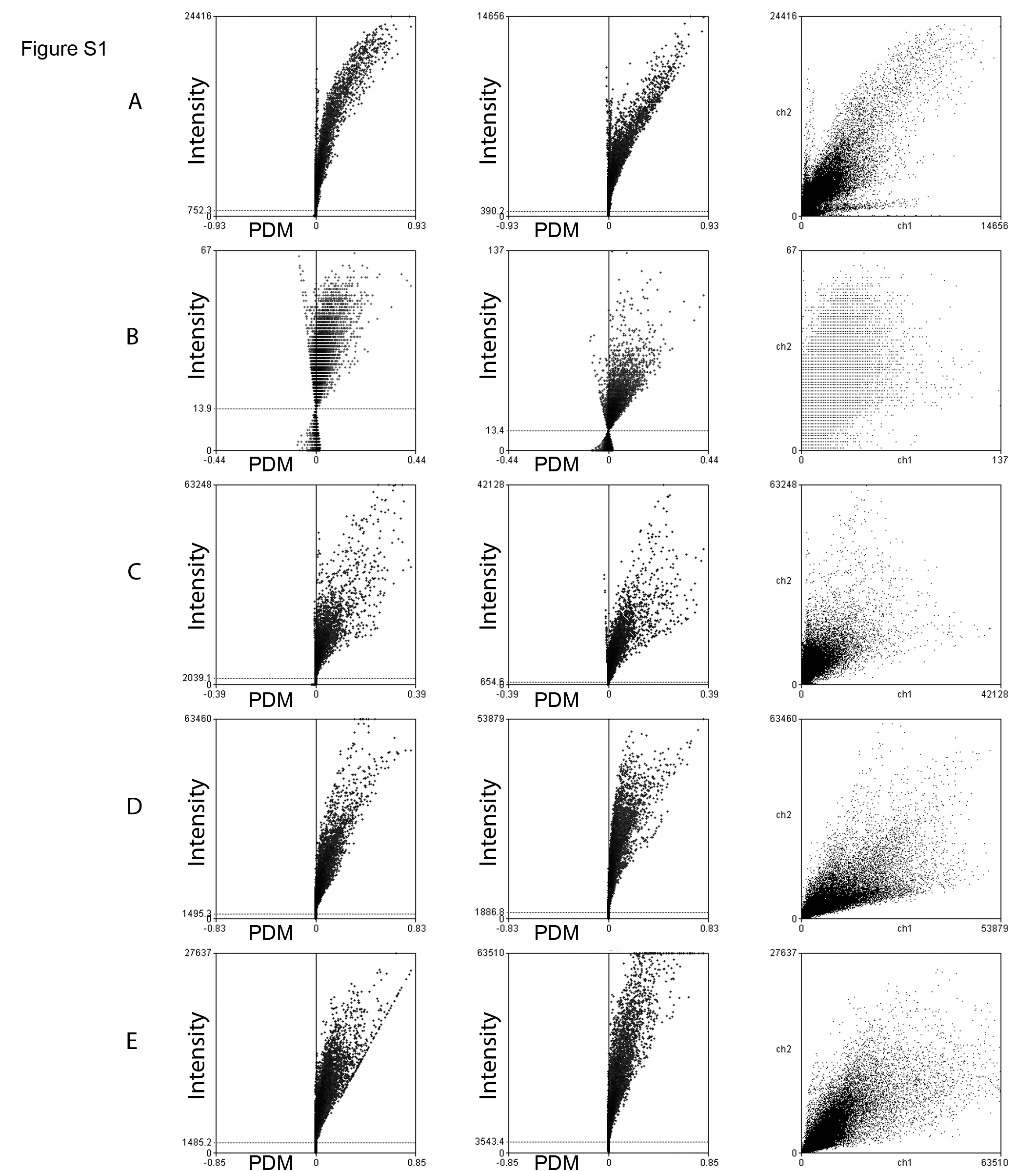

Supplement: Figure S1 — Scatter plots showing colocalization of fluorescent pixels from green (channel 1) and red (channel 2) channels in B16-F1 cells from the corresponding panels in Fig. 2 , quantified in table 1 . Left and middle columns contain graphs that plot pixel intensity (y-axis) versus the Product of the Differences from the Mean (PDM) value (x-axis) (see Methods). Positive PDM values correspond with colocalized pixels in both channels. Negative values indicate pixels that do not co-vary in their intensity in both channels and are not colocalized. The graphs in the right column plot pixel intensity of channel 1 versus pixel intensity of channel 2 as an additional means of relating the brightness data in both channels. The data in these graphs are not normalized. (TIF) [file pone.0043465.s001.tif]

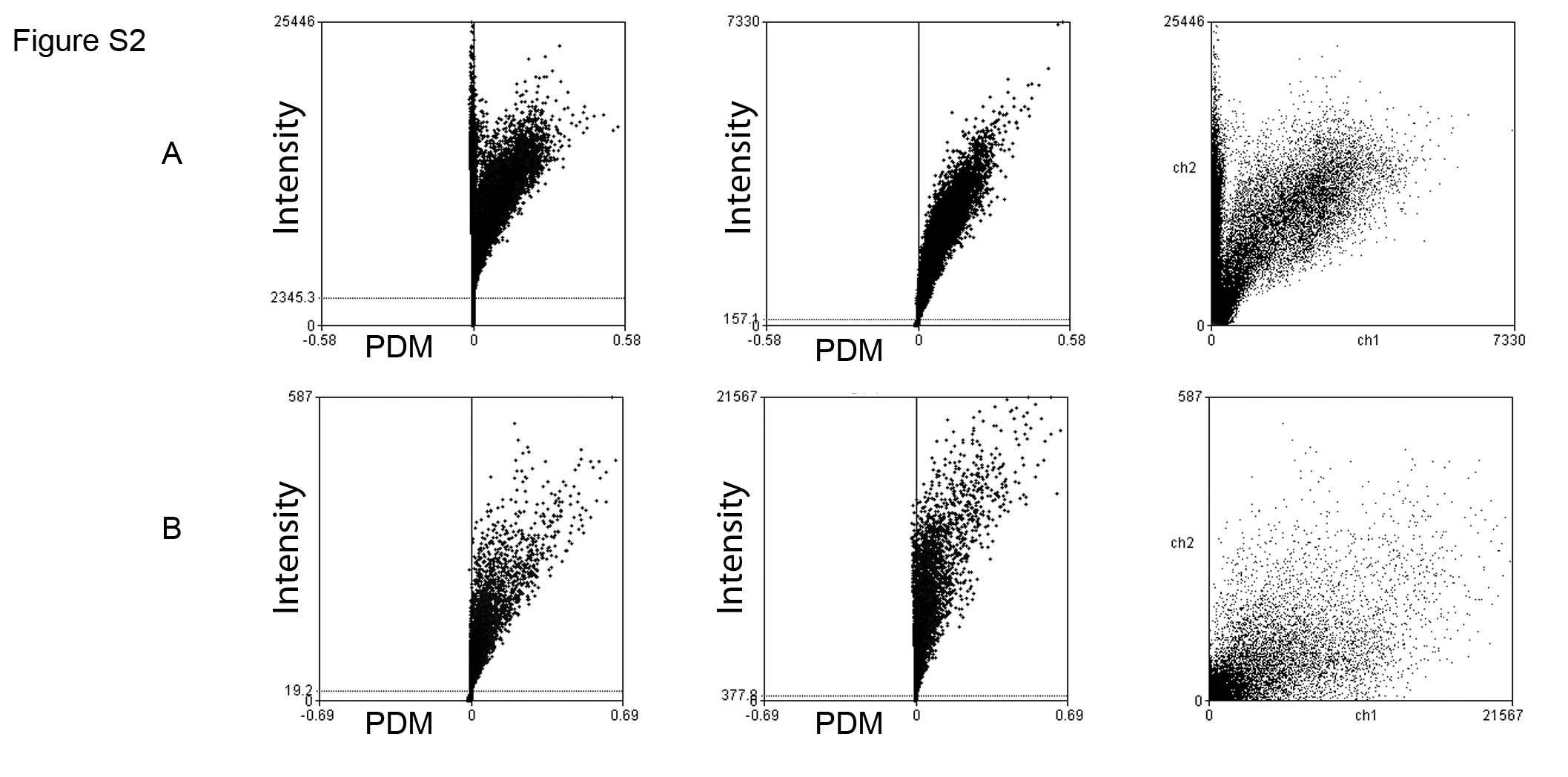

Supplement: Figure S2 — Scatter plots showing colocalization of fluorescent pixels from red and green channels in HEMs from corresponding sub-figures in Fig. 3 , quantified in the text. Same setup, procedure, and layout were used as described in S1. (TIF) [file pone.0043465.s002.tif]
